# Anteriolateral versus anterior–posterior electrodes in external cardioversion of atrial fibrillation: A systematic review and meta‐analysis of clinical trials

**DOI:** 10.1002/clc.23987

**Published:** 2023-02-09

**Authors:** Karam R. Motawea, Mostafa R. Mostafa, Merna Aboelenein, Mohamed Magdi, Hager Fathy, Sarya Swed, Mohamed M. Belal, Dina M. Awad, Rowan H. Elhalag, Nesreen E. Talat, Samah S. Rozan, Abdulqadir J. Nashwan, Naim Battikh, Bisher Sawaf, Mhd K. Albuni, Elias Battikh, Gihan M. Mohamed, Amr Farwati, Hani Aiash

**Affiliations:** ^1^ Faculty of Medicine, Alexandria University Alexandria Egypt; ^2^ Department of Medicine Rochester Regional Health/Unity Hospital Rochester New York USA; ^3^ Faculty of Medicine, Minia University Minya Egypt; ^4^ Faculty of Medicine, Aleppo University Aleppo Syria; ^5^ Nursing Department Hamad Medical Corporation Doha Qatar; ^6^ John H. Stroger, Jr. Hospital of Cook County Chicago Illinois USA; ^7^ Department of Internal Medicine Hamad Medical Corporation Doha Qatar; ^8^ Cardiovascular Perfusion Department Upstate Medical University Syracuse New York USA

**Keywords:** anterior–lateral electrode, anterior–posterior electrode, atrial fibrillation, cardioversion

## Abstract

The efficacy of anteriolateral versus anterior–posterior electrode positions in the success of atrial fibrillation's (AF) electrical cardioversion is unclear. Our aim is to perform a meta‐analysis to compare the success rate of both electrode positions. PUBMED, WOS, OVID, and SCOPUS were searched. Inclusion criteria were clinical trials that compared anterior–lateral with anterior–posterior electrodes in external cardioversion of AF. After the full‐text screening, 11 trials were included in the analysis. The total number of patients included in the study is 1845. The pooled analysis showed a statistically significant association between anterior–lateral electrode and increased cardioversion rate of AF (odds ratio [OR] = 1.40, 95% confidence interval [CI] = 1.02–1.92, *p* = .04). Subgroup analysis revealed a statistically significant association between the anterior–lateral electrode and increased cardioversion rate of AF in subgroups of less than five shocks, patients with 60 years old or more and patients with left atrial (LA) diameter >45 mm (OR = 1.72, 95% CI = 1.17–2.54, *p* = .006), (OR = 1.73, 95% CI = 1.18–2.54, *p* = .005), and (OR = 1.86, 95% CI = 1.04–3.34, *p* = .04), respectively. Anteriolateral electrode is more effective than anterior–posterior electrode in external cardioversion of AF, particularly in patients who have received less than 5 shocks, are 60 years old or older and have a LA diameter greater than 45 mm.

## Graphical abstract

Anterior–lateral versus anterior–posterior electrodes in external cardioversion of atrial fibrillation. A meta‐analysis

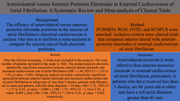

## 1 INTRODUCTION

Atrial fibrillation (AF) is the most common arrhythmia worldwide. The global burden of AF has increased significantly due to the aging population. By 2030, the United States may have over 12 million people with AF.^1^ AF has significant morbidity, linked to a threefold increased risk of heart fail­ure, a fivefold increased risk of stroke, and a 1.5–1.9‐fold increased chance of death. Rate or rhythm control, as well as anticoagulation to prevent thromboembolic complications, are the two basic techniques of controlling AF. The rate control strategy utilizes atrioventricular nodal blocking agents and long‐term anticoagulation. In the rhythm control strategy, sinus rhythm is restored through either pharmacological, electrical, or catheter ablation. Rate control was the primary strategy over the last decade based on the data from the AFFIRM trial that showed no survival benefit from the rhythm control over the rate control.^2^ However, recent data suggest that rhythm control, especially early in the disease, has fewer adverse cardiovascular outcomes.^3^

Electrical cardioversion is the most popular method of rhythm control in cardiology practice. It effectively terminates the AF in more than 90% of the cases.^4^ Electrical cardioversion can be safely done during the first 48 hours where the risk of clot formation is low.^5^ If the duration is more than 48 hours or unknown, a transthoracic echocardiogram is warranted to exclude atrial thrombus or systemic anticoagulation for 3 weeks before and 4 weeks after the cardioversion.^5^ Successful electrical cardioversion depends on proper patient selection, amount of electrical current delivery, number of shocks delivered, electrode size, and electrodeposition.^6^ The most common positions in practice are anterior–posterior and anterior–lateral. The evidence comparing their efficacy is uncertain. Some evidence suggests that the anterior–posterior posture is optimal for the external cardioversion of AF.^7^ On the other hand, others have shown no difference in cardioversion success between the two positions.^8^ As a result, we conducted a meta‐analysis of trials to see how the electrodeposition affects cardioversion success.

## 2 METHODS

Ethical approval is not required as this study is a meta‐analysis of published clinical trials. The present meta‐analysis was performed in accordance with Preferred Reporting Items for Systematic Reviews and Meta‐Analyses (PRISMA) guidelines and the Cochrane handbook.^9^

We systematically searched PUBMED, Web of Science, OVID, and SCOPUS from inception to January 30, 2022. The search included the following key terms: “Anterior lateral” OR “anterolateral” AND “Electrode” AND “Anterior posterior” OR “Anteroposterior” AND “atrial fibrillation” OR “AF”. We further reviewed the reference list of articles included in this review to include other relevant studies. Primary source studies published in peer‐reviewed journals were eligible for inclusion if they met the below criteria.

### 2.1 Eligibility criteria

Clinical trials that compared anterior–lateral electrode with anterior–posterior electrode in external cardioversion of AF. We excluded cohort studies, case reports, editorials, conference abstracts, and animal studies. And thus, the PICO criteria for our meta‐analysis will be:

Population: Patients with AF.

Intervention: Anterior–lateral electrode.

Comparison: Anterior–posterior electrode.

Outcome: Cardioversion success rate between anterior–lateral electrodeposition group and the anterior–posterior group.

### 2.2 Screening, data extraction, and risk of bias

Initial title and abstract screening were conducted by two reviewers (S. S. and H. F.) and all disagreements were discussed to reach a consensus, otherwise, a third opinion from (K. R. M.) was obtained.

Potentially eligible articles were imported for full‐text review and assessed for inclusion. We extracted data using an Excel sheet. Examples of data collected are study arms, number of patients in each group, age, sex (*n*), other baseline diseases, duration of AF, and baseline treatment.

We used the Cochrane tool (Risk of Bias 2) to assess the risk of bias in randomized trials. The following items were assessed (overall bias, selection of the reported result, measurement of the outcome, missing outcome data, deviations from intended interventions, and randomization process).

### 2.3 Data analysis

We used the Review Manager Software version 5.4 to perform the meta‐analysis; the dichotomous outcomes were presented as odds ratios (OR) with a 95% confidence interval. In case of heterogeneity (*χ* ^2^ *p* < .05), a random effect model was used otherwise, a fixed‐effect model was employed, in general; the results were considered significant if the *p* value was less than .05.

## 3 RESULTS

### 3.1 Summary of studies

**Figure 1:**
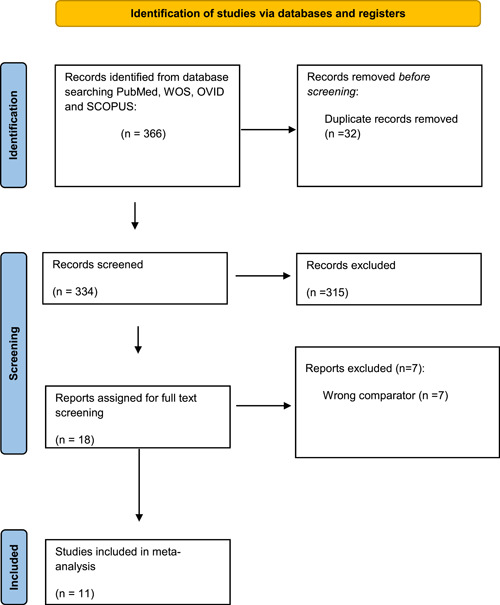
Preferred Reporting Items for Systematic Reviews and Meta‐Analyses flow diagram.

After a comprehensive search of the literature, 366 studies resulted, and then became 334 were eligible for the title and abstract screening after the removal of duplicates. Of the 334, 315 were irrelevant and 19 studies were eligible for full‐text screening. Finally, 11 clinical trials were included in the meta‐analysis after the full‐text screening, as shown in the PRISMA in (Figure 1), summary of the included studies is shown in Table 1.

**Table 1:** Summary of the included studies.

ID	Study arms		Number of patients in each group		Age (Years)		sex (n)		
	group 1	group 2	group 1	group 2	group 1	group 2	group 1		group 2
							female	male	female
**Alp 2000**	Antero‐lateral	Antero‐posterior	30	29	Mean = 67.8, SD = 8.1	Mean = 66.8, SD = 7.9	11 (37%)	19 (63%)	7 (24%)
**CHEN 2003**	Antero‐posterior	Antero‐lateral	39	31	Mean = 57.6, SD = 10.1	Mean = 59.1, SD = 14.7	27 (69%)	12 (31%)	11 (35.5%)
**Walsh 2005**	Antero‐lateral	Antero‐posterior	150	157	Mean = 67, SD = 10	Mean = 66, SD = 14	55 (37%)	95 (63%)	57 (36%)
**Kirchhof 2002**	Antero‐posterior	Antero‐lateral	52	56	Mean = 62, SD = 2	Mean = 58, SD = 2	14 (27%)	38 (73%)	12 (21%)
**Schmidt 2012**	Antero‐lateral	Antero‐posterior	233	234	Mean = 68.7, SD = 9.5	Mean = 68.9, SD = 9.3	77 (33%)	156 (67%)	76 (32%)
**Stiell2020**	Anterolateral	Anteroposterior	127	117	Drug shock,mean=60,SD = 15·1 (years) Shock only,mean=60·1,SD = 14·8 (years)				Drug shock M/F 134/75 Shock only M/F 126/66
**Mathew 1999**	Antero‐lateral	Antero‐posterior	45	45	Mean = 65.5, SD = 10		Female = 30 (33%), Male = 60 (67%)		
Botto 1999	Anterolateral	Anteroposterior	151	150	62	62	57	94	61
**Siaplaouras 2005**	Anterolateral	Anteroposterior	63	60	66 ± 10	67 ± 10	25%	75%	33%
Vogiatzis 2009	anterolateral	Anteroposterior	32	30	Mean=60.1,SD = 8.6	Mean=61.6,SD = 7.2	11	21	10
**Kerber1981**	Anterolateral	Anteroposterior	58	53	Mean=56,SD = 15	Mean=57,SD = 13	19	39	14

	other baseline diseases			Duration of atrial fibrillation		Treatment at baseline		Direct current cardioversion	
ID	Male	group 1	group 2	group 1	group 2	group 1	group 2		Patches
**Alp 2000**	22 (76%)	Ischaemic heart disease 6 (20%) Hypertension 5 (25%) Valvular heart disease 3 (10%) Thyrotoxicosis 1 (3%)	Ischaemic heart disease 3 (10%) Hypertension 11 (38%) Valvular heart disease 1 (3%) Thyrotoxicosis 1 (3%)	Mean = 23, Range = 2–104 (weeks)	Mean = 31, Range = 8–104 (weeks)	Amiodarone 8 (27%) Flecainide 14 (47%) Sotalol 1 (3%) Verapamil 2 (7%) Digoxin 15 (50%)	Amiodarone 6 (21%) Flecainide 16 (55%) Sotalol 1 (3%) Verapamil 0 Digoxin 11 (38%)	360 J (2 shocks)	Fresh gel pads (11*15 cm)
**CHEN 2003**	20 (64.5%)	Valvular heart disease 18 (46.2%) Hypertension 15 (38.5%) Rheumatic heart disease 11 (28.2%) Idiopathic cardiomyopathy 7 (17.9%) Coronary artery disease 3 (7.7%) Lone atrial fibrillation 1 (2.7%)	Valvular heart disease 11 (35.5%) Hypertension 10 (32.3%) Rheumatic heart disease 8 (25.8%) Idiopathic cardiomyopathy 5 (16.1%) Coronary artery disease 1 (3.2%) Lone atrial fibrillation 5 (16.1%)	Mean = 25.9, SD = 33.3 (months)	Mean =23.8, SD = 38.6 (months)	Amiodarone 25 (64.1%) Beta‐blockers 25 (64.1%) Calcium channel blockers 7 (17.9%)	Amiodarone 18 (58.1%) Beta‐blockers 7 (22.6%) Calcium channel blockers 4 (12.9%)	initial 100 joules (J) and then increased to 150 J, 200 J, 300 J, and 360 J if needed.	self‐adhesive, pre applied, low impedance, disposable electrode pads (R2 Medical System Inc, Carlsbad, California, USA)
**Walsh 2005**	100 (64%)	Ischaemic heart disease 47 (31%) Hypertension 57 (38%) Valvular heart disease 36 (24%) Alcohol excess 13 (9%) Lone AF 21 (14%)	Ischaemic heart disease 61 (39%) Hypertension 81 (52%) Valvular heart disease 26 (17%) Alcohol excess 8 (5%) Lone AF 15(10%)	Mean = 19, SD = 33 (weeks)	Mean = 26, SD = 48 (weeks)	Digoxin 63 (42%) B‐Blocker excluding sotalol 89 (59%) Rate limiting calcium antagonist 7(5%) Sotalol 5 (35%) Amiodarone 14 (9%) Flecainide 3 (2%) Propafenone 10 (7%) Sedation Midazolam 100 (67%) Sedation Propofol 49 (33%)	Digoxin 61 (39%) B‐Blocker excluding sotalol 93 (59%) Rate limiting calciumantagonist 5 (3%) Sotalol 6 (4%) Amiodarone 15 (10%) Flecainide 3 (2%) Propafenone 10 (6%) Sedation Midazolam 106 (68%) Sedation Propofol 52 (33%)	Shocks were delivered at 70, 100, 150, and 200 J. If the fourth shock was unsuccessful, the pads were switched to the alternative position (crossover) and a further 200 J shock was attempted.	self‐adhesive electrode pads (Agilent Adult Plus Electrode pads ref: M3713A)
**Kirchhof 2002**	44 (79%)	Coronary‐artery disease 13 (25%) Dilated cardiomyopathy 7 (13%) Hypertrophic cardiomyopathy 3 (6%) Conduction disturbance 6 (12%) Myocarditis 1 (2%) Hypertension (blood pressure >140/90 mm Hg) 29 (56%) Previous stroke or transient neurological deficit 5 (10%) Atrial or ventricular septal defect 3 (6%) Lone atrial fibrillation 14 (23%)	Coronary‐artery disease 14 (25%) Dilated cardiomyopathy 13 (23%) Hypertrophic cardiomyopathy 3 (5%) Conduction disturbance 2 (4%) Myocarditis 0 Hypertension (blood pressure >140/90 mm Hg)22 (39%) Previous stroke or transient neurological deficit 2 (4%) Atrial or ventricular septal defect 2 (4%) Lone atrial fibrillation15 (27%)	Median = 5, Range = 0·1–120 (months)	Median = 4, Range = 0·1–120 (months)	Sodium‐channel blocker 7 (13%) B‐blocker 26 (50%) Sotalol 10 (19%) Calcium antagonist 4 (8%) Digitoxin 36 (69%) Amiodarone 8 (15%)	Sodium‐channel blocker 9 (16%) B‐blocker 32 (57%) Sotalol 14 (25%) Calcium antagonist 4 (7%) Digitoxin 35 (63%) Amiodarone 12 (21%)	50 J, 100 J, 200 J,300 J and 360 J	hand‐held sintered steel electrodes covered by conducting gel
**Schmidt 2012**	158 (68%)	Arterial hypertension 149 (64%) Heart failure 67 (29%) Valvular heart disease 26 (11%) Ischemic heart disease 28 (12%) Diabetes 23 (10%) Previous stroke or transient ischemic attack 21 (9%)	Arterial hypertension 151 (65%) Heart failure 54 (23%) Valvular heart disease 33 (14%) Ischemic heart disease 27 (12%) Diabetes 22 (9%) Previous stroke or transient ischemic attack 17 (7%)	Median = 9, IQR = 1–60 (months)	Median = 5, IQR = 1–46 (months)	Amiodarone 39 (17%) Digoxin 42 (18%) Flecainide 4 (2%) β‐blockers 194 (83%) ACE inhibitor or angiotensin II receptor blocker 123 (53%)	Amiodarone 30 (13%) Digoxin 32 (14%) Flecainide 2 (%1) β‐blockers 179 (76%) ACE inhibitor or angiotensin II receptor blocker 114 (49%)	f 100 J, 150 J, 200 J, and 360j	self‐adhesive gel electrodes.
**Stiell2020**	Drug shock Congestive heart failure 0 (0%) Acute coronary syndrome 1 (1%) Shock only Congestive heart failure 2 (1%) Acute coronary syndrome 1 (1%)			Duration (mean h) Drug shock 14·3 (20·6) shock only 16·8 (25·8		Drug shock: Anticoagulants 66 (32%), Novel anticoagulants 51 (25%),Warfarin 15(7%),Antiarrhythmics 13 (6%),Amiodarone 9 (4%),Propafenone 3 (2%),Flecainide 1 (1%),Sotalol 0 (0%),Antiplatelet agents 59 (29%),Aspirin 55 (27%),Clopidogrel 4 (2%),Cardiac medications 101 (50%),β blocker 75 (37%),Calcium channel blocker 26 (13%). Shock only: Anticoagulants 66 (34%),Novel anticoagulants 52(27%),Warfarin 14(7%),Antiarrhythmics 13 (7%),Amiodarone 9 (5%),Propafenone 0(0%),Flecainide 1 (1%),Sotalol 1 (1%),Antiplatelet agents 48 (25%),Aspirin 46 (24%),Clopidogrel 2(1%),Cardiac medications 83 (43%),β blocker 61 (32%),Calcium channel blocker 22 (12%).		up to three shocks, each of ≥200 J	standard adhesive pads
**Mathew 1999**	Ischaemic heart disease 28 (31%) Hypertensive heart disease 19 (21%) Rheumatic heart disease 15 (16%)			Mean = 14.7, SD = 40.2 (months)		NA		100 J, 200 J, 300 J, and 360 J	self adhesive pads
Botto 1999	89	Systemic hypertension41 (27%) Valvar heart disease 42 (28%) Coronary artery disease 14 (9%) Cardiomyopathy 15 (10%) Previous AF episodes 75 (50%)	Systemic hypertension 40 (27%) Valvar heart disease 43 (29%) Coronary artery disease 18 (12%) Cardiomyopathy 17 (11%) Previous AF episod 75 (50%)	84 (92)	92 (96)	Quinidine 2 (1%) Propafenone 25 (17%) Flecainide 3 (2%) Amiodarone 62 (41%) Sotalol 7 (5%)	Quinidine 3 (2%) Propafenone 18 (12%) Flecainide 1 (1%) Amiodarone 69 (46%) Sotalol 6 (4%)	a 3 J/kg body weight dc shock, then a 4 J/kg shock (maximum 360 J), and finally a second 4 J/kg shock using the alternative paddle location.	two self adhesive, preapplied, low impedance, disposable patch electrodes (R2 Medical System Inc, Carlsbad, California, USA).
**Siaplaouras 2005**	67%	Arterial hypertension [n (%)] 18 (28) Valvular disease [n (%)] 11 (18) Coronary artery disease [n (%)] 16 (25) Cardiomyopathy [n (%)] 11 (17)	Arterial hypertension [n (%)] 26 (44) Valvular disease [n (%)] 14 (23) Coronary artery disease [n (%)] 10 (16) Cardiomyopathy [n (%)] 3 (5)	3.8 ± 9	3.0 ± 5	Class I [n (%)] 3 (5) Class II [n (%)] 19 (30) Sotalol [n (%)] 13 (21) Amiodarone [n (%)] 19 (30) Class IV [n (%)] 1 (2) Digitalis [n (%)]4 (6)	Class I [n (%)] 3 (5) Class II [n (%)] 29 (48) Sotalol [n (%)] 9 (15) Amiodarone [n (%)] 16 (27) Class IV [n (%)] 0 (0) Digitalis [n (%)] 2 (3)	(120‐150‐200‐200 W).	self‐adhesive CV pads (Pro‐padz biphasic, Zoll Medical Corp, Burlington, Mass, USA)
Vogiatzis 2009	20	Coronary artery disease n (%) 4 (12.5%) Hypertension n (%) 4 (12.5%) Valvular heart disease n (%) 6 (18.8%) Dilated cardiomyopathy n (%) 2 (6.3%) Lone atrial fibrillation n (%) 16 (50.0%)	Coronary artery disease n (%) 6 (20.0%) Hypertension n (%) 4 (13.5%) Valvular heart disease n (%) 5 (16.7%) Dilated cardiomyopathy n (%) 2 (6.7%) Lone atrial fibrillation n (%) 18 (60.0%)	49.13 ± 21.84	51.25 ± 13.75	Digoxin n (%) 18 (56.3%) Beta‐blockers n (%) 16 (50.0%) Calcium channels blockers n (% 8 (25.0%)	Digoxin n (%) 14 (46.7%) 0.5 Beta‐blockers n (%) 15 (50.0%) Calcium channels blockers n 5 (16.7%)	200 J, 300 J and 360 J	two self‐adhesive, preapplied, low impedance, disposable patch electrodes (R2 Ballard Medical Products, Draper, Utah, USA) of 12 cm diameter.
**Kerber1981**	39	Congestive heart failure 5 Chronic obsructive bulmonary disease 7 Mitral stenosis or regurgitation(or both) 20 Aortic stenosis or regurgitation(or both) 11 Coronary artery disease 25	Congestive heart failure 8 Chronic obsructive bulmonary disease 8 Mitral stenosis or regurgitation(or both) 19 Aortic stenosis or regurgitation(or both) 12 Coronary artery disease 22	51 ± 117	46 ± 111	Digitalis 37 Quinidine 16 Propranolol 9	Digitalis 31 Quinidine 10 Propranolol 9	100 J,200 J,300 J,400 J and 460 J	The paddles were coated with a layer of Hewlett packard redux paste

Abbreviations: ACE, angiotensin‐converting enzyme; IQR, interquartile range; NA, not available.

**Figure 2:**
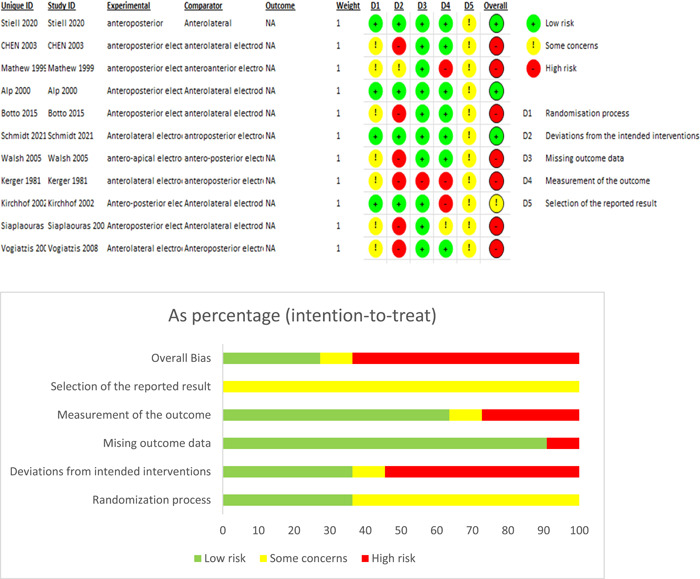
Risk of bias assessment. NA, not available.

The cardioversion rate was compared between anterior–lateral and anterior–posterior electrode positions in the 11 studies. Subgroup analysis was done according to the number of shocks, age, and left atrial (LA) diameter. The number of shocks subgroup was divided into two subgroups: five shocks or less than five shocks. The age subgroup was divided into two subgroups: 60 years old and more or less than 60 years old. The LA diameter subgroup was divided into two subgroups: more than 45 mm or 45 mm and less than 45 mm. The overall risk of bias was high in most of the studies, as we found significant bias in deviation from the intended interventions domain in six studies, missing outcome data domain in one study, and measurement of the outcome domain in three studies; however, we found no significant bias in randomization process domain in the included studies as shown in Figure 2.

The total number of patients included in the study is 1845 patients, 931 patients in the anterior–lateral electrode group, and 941 patients in the anterior–posterior electrode group, other baseline data are shown in Table 2.

**Table 2:** Baseline characteristics of the included studies.

ID	Study design	Country of the study	Study arms	Outcomes	Conclusion
Alp 2000	Randomized controlled trial	The United Kingdom	Group 1: anterolateral (AL) Group 2: Anteroposterior (AP)	In the first 360‐J DC shock, a significantly greater proportion of patients were restored to sinus rhythm from the AL position (18/30) compared to the AP position (10/29) (P50.048). For those patients remaining in atrial fibrillation(AF), there was no difference in the proportions cardioverted from the AL position (4/19) compared to the AP position (5/12) with the second 360 J DC shock (P50.22). The total cardioversion (CV) success rate was 23/30 (77%) for AL followed by AP shocks compared to a success rate of 14/29 (48%) for AP followed by AL shocks, and this difference was significant (P50.024). There was no significant difference in the mean energy delivered for patients randomized to an initial AL shock (504 J), compared to patients given an initial AP shock (583 J) (P50.1).	The study concluded that the AL paddle position appears more effective for DC CV of persistent AF.
Chen 2003	Randomized controlled trial		Group 1: AP Group 2: AL	Mean transthoracic impedance (TTI) was significantly lower in the AP group than in the AL group. However, the cumulative success rates at each energy level were similar in the two groups (23% vs. 19.4%, 41% vs. 45.2%, 66.7% vs. 74.2%, 79.5% vs. 77.4%, and 84.6% vs. 83.9% at 100 J, 150 J, 200 J, 300 J, and 360 J, respectively). In the AP group, converters showed slightly lower TTI compared to nonconverters. In the AL group, converters showed significantly lower TTI compared to nonconverters. However, for all patients as a group, TTI was the only predictor for CV success and showed a significant relationship to the energy required for CV, which can be described by a quadratic equation. Rather than pad position, TTI is the single factor that significantly affects CV and correlates with energy requirement.	The study concluded that the relationship between energy requirement and TTI further allows the estimation of energy requirements to achieve a successful CV.
Walsh 2005	Randomized controlled trial	The United Kingdom	Group 1: AL Group 2: AP	Shock 1 was successful in 54/150 (36%) anteroanterior (AA) and 45/144 (31%) AP patients, whereas success was achieved by shock 2 in 99/150 (66%) AA and 74/144 (51%) AP, by shock 3 in 123/150 (82%) AA and 109/144 (76%) AP, and by shock 4 in 143/150 (95%) AA and 127/144 (88%) AP and after cross‐over in 144/150 (96%) AA and 135/144 (94%) AP. The overall success rate was higher than expected at 95%. Pad position was not associated significantly with success. There was a trend toward an improved outcome with the AA configuration (*p* 1⁄4 .05).	The study concluded that the influence of pad position for DCC of AF may be less pertinent with ICB waveforms than with monophasic waveforms.
Kirchhof 2002	Randomized controlled trial	Germany	Group 1: AP Group 2: AL	CV was successful in a higher proportion of the AP than the AL group (50 of 52 [96%] vs. 44 of 56 [78%]), the difference of 23.7% (95% CI: 9.1–37.8, *p* = .009). Cross‐over from the AL to the AP electrodeposition was successful in 8 of 12 patients, whereas cross‐over in the other direction was not successful (2 patients). After cross‐over, CV was successful in 102 of 108 randomized patients (94%).	The study concluded that an AP electrodeposition is more effective than the AL position for the external CV of persistent AF. These results should be considered in clinical practice, for the design of defibrillation electrode pads, and when guidelines for the CV of AF are updated.
Schmidt 2012	Randomized controlled trial	Denmark	Group 1: AL Group 2: AP	The primary outcome occurred in 126 patients (54%) assigned to the AL electrodeposition and in 77 patients (33%) assigned to the AP electrodeposition (risk difference, 22 percentage points [95% CI: 13–30]; *p* < .001). The number of patients in sinus rhythm after the final CV shock was 216 (93%) assigned to AL electrode positioning and 200 (85%) assigned to AP electrode positioning (risk difference, 7 percentage poin**t**s [95% CI: 2–12]). There were no significant differences between groups in any safety outcomes.	The study concluded that AL electrode positioning was more effective than AP electrode positioning for the biphasic CV of AF. There were no significant differences in any safety outcome.
Stiell 2020	Randomized controlled trial	Canada	Group 1: AL Group 2: AP	In the drug–shock group (*n* = 204), conversion to sinus rhythm occurred in 196 (96%) patients, and in the shock‐only group (*n* = 192), conversion occurred in 176 (92%) patients (absolute difference of 4%; 95% CI: 0–9; *p* = .07). The proportion of patients discharged home was 97% (*n* = 198) versus 95% (*n* = 183; *p* = .60). A total of 106 (52%) patients in the drug–shock group converted after drug infusion only. No patients had serious adverse events in follow‐up. The different pad positions in Protocol 2 (*n* = 244), had similar conversions to sinus rhythm (119 [94%] of 127 in the AL group vs. 108 [92%] of 117 in AP group; *p* = .68).	The study concluded that both the drug–shock and shock‐only strategies were highly effective, rapid, and safe in restoring sinus rhythm for patients in the emergency department with acute AF, avoiding the need for a return to the hospital. The drug infusion worked for about half of the patients and avoided the resource‐intensive procedural sedation required for electrical CV. We also found no significant difference between the AL and AP pad positions for electrical CV. Immediate rhythm control for patients in the emergency department with acute AF leads to excellent outcomes.
Mathew 1999	Randomized controlled trial	The United Kingdom	Group 1: AA Group 2: AP	CV was successful in 81% of the patients (73/90). There was no statistically significant difference in the CV success rate (AA 84%, 38/45 patients; AP 78%, 35/45 patients; *p* = .42) or mean (SD) energy requirement for all patients (AA 223 [96.1] J; AP 232 [110] J) or for patients who were successfully cardioverted (AA 197.9 [82.4] J; AP 195.4 [97.2] J; *p* = .9) between the two pad positions. The mean TTI for the first shock (AA 77.5 [18.4] Ω; AP 73.7 [18.7] Ω; *p* = .34) was not significantly different between the two groups. TTI correlated significantly with body mass index, percentage body fat, and chest AP diameter. There was a progressive decrease in TTI with serial shocks. While etiology and TTI were the two independent significant predictive factors for energy requirement, the duration of AF was the only independent predictor of CV success in a multivariate analysis.	The study concluded that electrode pad position is not a determinant of CV success rate or energy requirement.
Botto 1999	Randomized controlled trial		Group 1: AL Group 2: AP	The two groups were comparable for all clinical variables evaluated. The cumulative percentage of patients successfully converted to sinus rhythm was 58% in group AL and 67% in group AP with low‐energy DC shock (NS); this rose to 76% in group AL and to 87% in group AP with high‐energy DC shock (*p* = .013). Thirty‐seven patients in group AL and 19 in group AP experienced DC shock with the alternative paddle position; AF persisted on 10/37 in group AL and in 10/19 in group AP. Mean DC shock energy requirements were lower for group AP patients than for group AL patients, at 383 (235) versus 451 (287) J, *p* = .025. Arrhythmia duration was the only factor that affected the technical success of external CV (successful: 281 patients, 80 (109) days; unsuccessful: 20 patients, 193 (229) days; *p* < .0001). The success rate was lower if AF persisted for >6 months: 29 of 37 (78%) versus 252 of 264 (95%); *p* = .0001.	The study concluded that an AP defibrillator paddle position is superior to an AL location with regard to technical success in the external CV of stable AF, and permits lower DC shock energy requirements. Arrhythmia duration is the only clinical variable that can limit the restoration of sinus rhythm.
Siaplaouras 2005	Randomized controlled trial	Germany	Group 1: AL Group 2: AP	Both groups (*N* = 123, mean age 66 years, 71% male, 83% with structural cardiovascular disease or hypertension) did not differ concerning age, sex, body mass index, chronic antiarrhythmic therapy, duration of AF, left ventricular ejection fraction, and left atrial diameter. Cumulative success rates were comparable (AP 94.9% vs. AL 95.2%, *p* = ns). First‐shock efficacy did not differ (AP 78.3% vs. AL 74.6%, *p* = ns). Early recurrent AF (AF relapse b1 min after successful CV) occurred in 8.1% (AP 11.6% vs. AL 4.8%, *p* = ns). The mean number of shocks was 1.3 per patient with the AP configuration and 1.4 per patient with the AL configuration (*p* = ns). Mean cumulative energy delivery was also comparable (AP 171 WS vs. AL 198 WS, *p* = ns).	The study concluded that both electrode positions are similar in the biphasic external CV of AF with regard to acute success and early recurrent AF. Also, the number of shocks needed and energy delivery is comparable with both electrode configurations.
Vogiatzis 2009	Randomized controlled trial	Greece	Group 1: AL Group 2: AP	After all shocks were delivered, there was no difference in the CV success rate between the two groups. However, a significantly greater proportion of patients in group B were restored to sinus rhythm after the second shock of 300 J was delivered (*p* = .005). Mean shock energy requirements and peak serum creatine kinase levels were lower for group B than for group A (*p* = .049 and *p* = .021, respectively). Troponin T serum levels were not increased after the CV attempts in either group.	The study concluded that an AP electrodeposition is more effective in achieving restoration of sinus rhythm in lower energy shock levels compared to the AL position.
Kerber 1981	Randomized controlled trial	United States	Group 1: AL Group 2: AP	Overall CV success rates with either paddle position were similar (greater than 90%). The larger paddles did not significantly reduce energy requirements for the CV of either arrhythmia. In AF, the majority of patients converted to normal sinus rhythm when given 100 or 200 J of energy. There were no significant differences in total success rates or in rates of success at any given energy level (i.e., energy requirements) when AP placement was compared with AP placement. When patients in each of these groups were subdivided into two smaller groups based on the size of the paddles, there still were no significant differences in overall success rates or energy requirements.	The study concluded that AL paddles are as effective as AP paddles for the elective CV of atrial arrhythmias and that there is no demonstrable advantage to using paddles that are larger than the standard size in either position (*N Engl J Med*. 1981; 305:658‐662).

Abbreviations: CI, confidence interval; DCC, direct current cardioversion; ICB, impedance compensated biphasic; ns, not significant; WS, watt‐hour per second.

### 3.2 Outcomes

#### 3.2.1 Overall cardioversion rate

**Figure 3:**
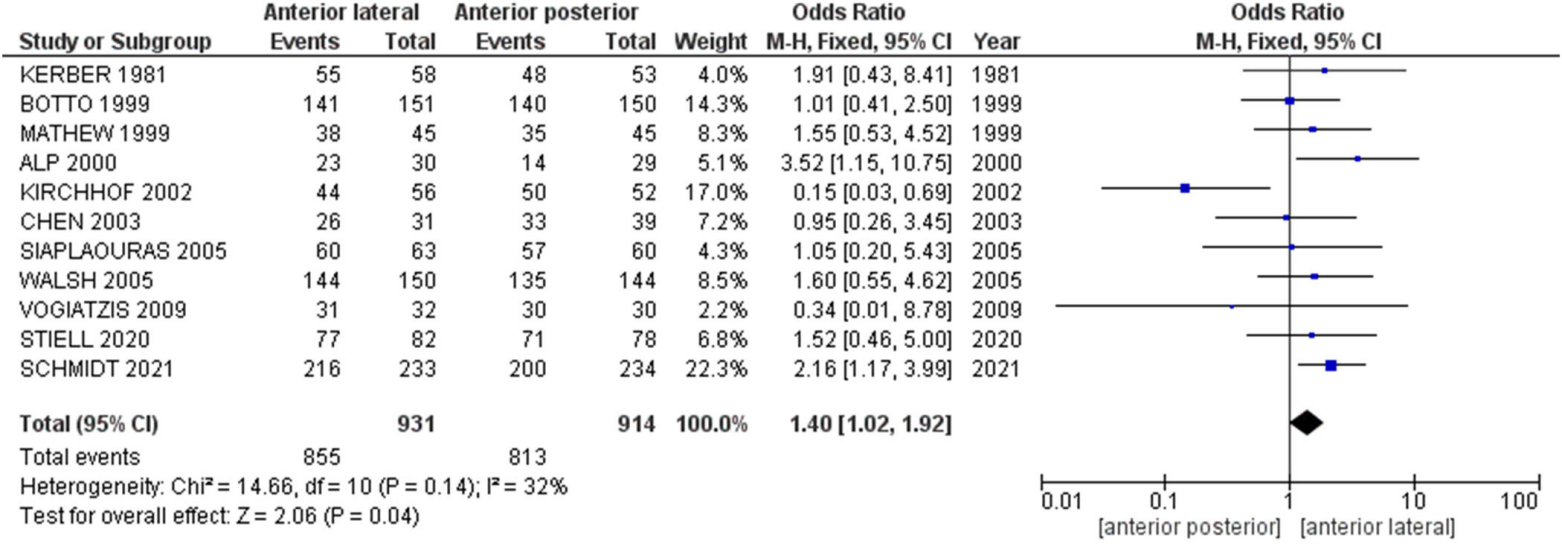
Forest plot of the comparison between the anterior–lateral electrode and anterior–posterior electrode in external cardioversion of atrial fibrillation in the overall analysis. CI, confidence interval; M–H, Mantel–Haenszel.

**Figure 4:**
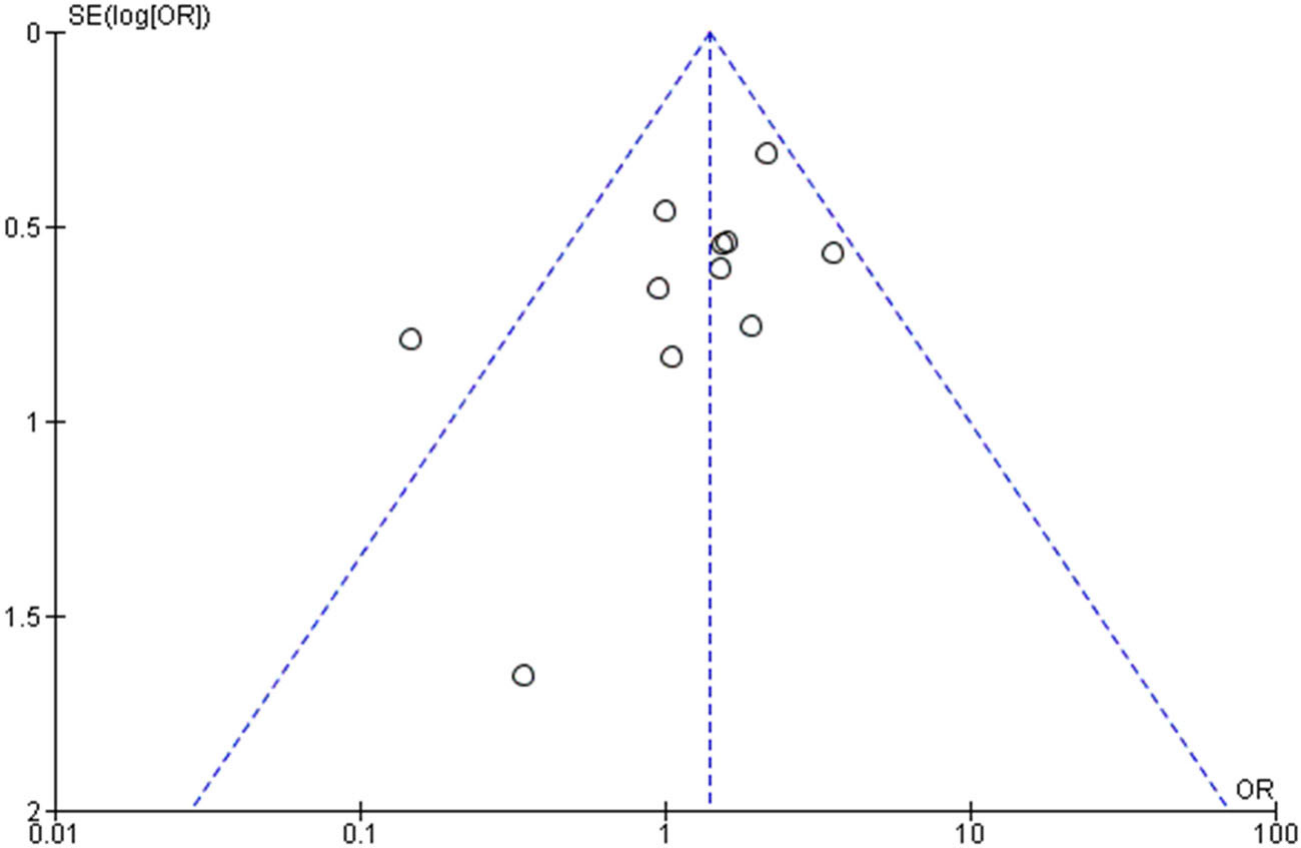
Publication bias. OR, odds ratio.

The pooled analysis showed a statistically significant association between the anterior–lateral electrode and increased cardioversion rate of AF compared with anterior–posterior electrode (OR = 1.40, 95% CI = 1.02–1.92, *p* = .04), indicating that anterior–lateral electrodeposition is better than anterior–posterior electrodeposition in the external cardioversion of AF. We observed no heterogeneity among studies (*p* = .14, *I* ^2^ = 32%), Figure 3. No publication bias was observed, as shown in Figure 4.

#### 3.2.2 Subgroup analysis

**Figure 5:**
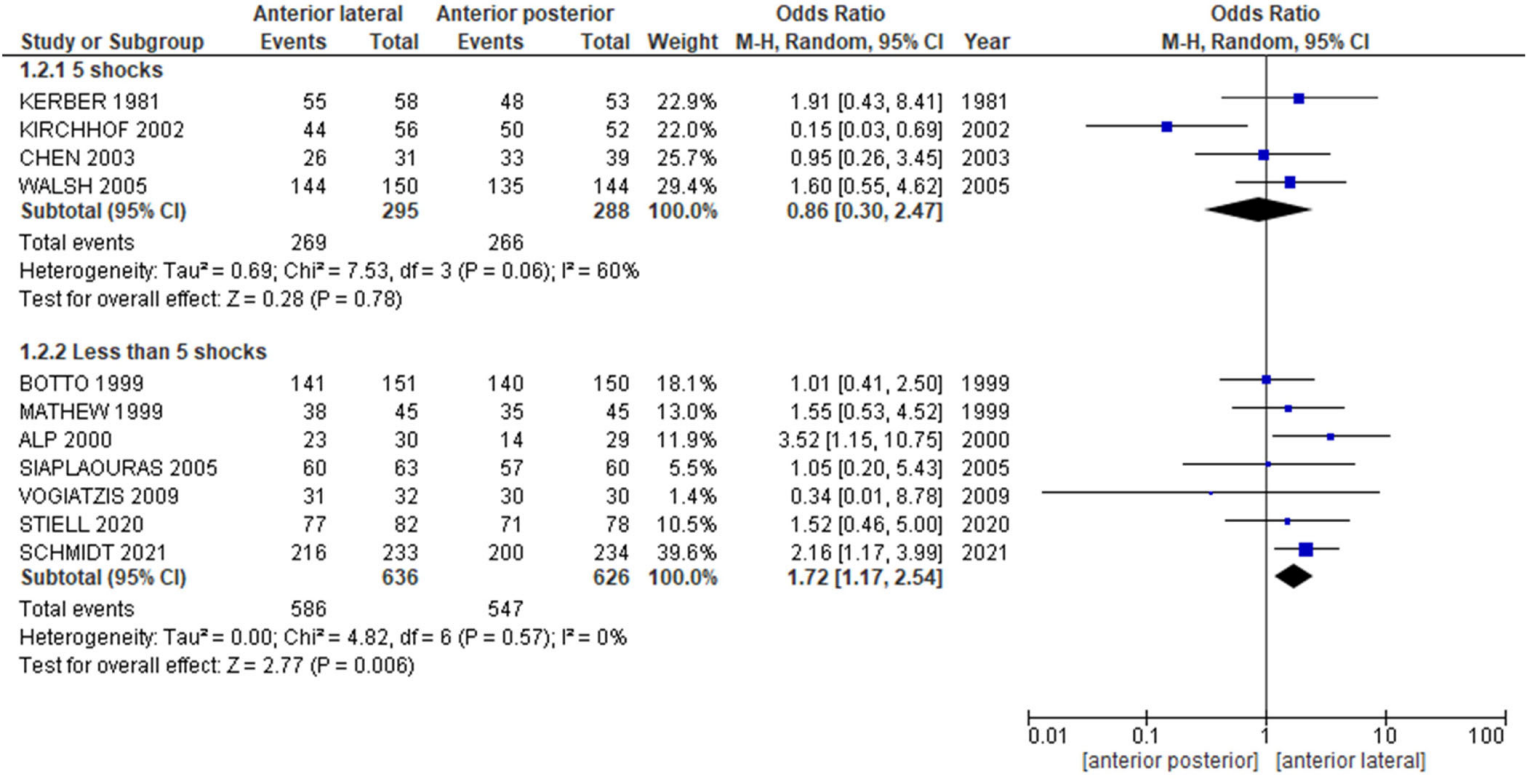
Number of shocks subgroup analysis. CI, confidence interval; M–H, Mantel–Haenszel.

*Number of shocks*: The pooled analysis showed no statistically significant difference between anterior–lateral electrode and anterior–posterior electrode in the subgroup of five shocks (OR = 0.86, 95% CI = 0.30–2.47, *p* = .78). We observed no significant heterogeneity among studies in this subgroup (*p* = .06, *I* ^2^ = 60%), Figure 5.

The pooled analysis showed a statistically significant association between anterior–lateral electrode and increased cardioversion rate of AF compared with anterior–posterior electrode in a subgroup of less than 5 shocks (OR = 1.72, 95% CI = 1.17–2.54, *p* = .006). We observed no significant heterogeneity among studies in this subgroup (*p* = .57, *I* ^2^ = 0%), Figure 5.

*Age*: The pooled analysis showed a statistically significant association between anterior–lateral electrode and increased cardioversion rate of AF compared with anterior–posterior electrode in a subgroup of 60 years old and more (OR = 1.73, 95% CI = 1.18–2.54, *p* = .005). We observed no significant heterogeneity among studies in this subgroup (*p* = .57, *I* ^2^ = 0%), Supporting Information: Figure 1.

The pooled analysis showed no statistically significant difference between anterior–lateral electrode and anterior–posterior electrode in a subgroup of less than 60 years old (OR = 0.66, 95% CI = 0.16–2.78, *p* = .57). We observed a significant heterogeneity among studies in this subgroup (*p* = .05, *I* ^2^ = 67%), Supporting Information: Figure 1. We performed the leave‐one‐out test by removing^7^ study, and the heterogeneity was solved (*p* = .48, *I* ^2^ = 0%), and the pooled analysis showed no difference between the two groups (OR = 1.28, 95% CI = 0.48–3.40, *p* = .62).

*LA diameter*: The pooled analysis showed no statistically significant difference between anterior–lateral electrode and anterior–posterior electrode in a subgroup of more than 45 mm (OR = 1.18, 95% CI = 0.46–3.00, *p* = .73). We observed a significant heterogeneity among studies in this subgroup (*p* = .02, *I* ^2^ = 64%), Supporting Information: Figure 2. We performed a leave‐one‐out test by removing^7^ study and the heterogeneity was solved (*p* = .59, *I* ^2^ = 0%), and the pooled analysis showed a statistically significant association between the anterior–lateral electrode and increased cardioversion rate of AF compared with anterior–posterior electrode in a subgroup of more than 45 mm (OR = 1.86, 95% CI = 1.04–3.34, *p* = .04).

The pooled analysis showed no statistically significant difference between anterior–lateral electrode and anterior–posterior electrode in a subgroup of 45 mm and less than 45 mm (OR = 1.07, 95% CI = 0.56– 2.06, *p* = .83). We observed no significant heterogeneity among studies in this subgroup (*p* = .77, *I* ^2^ = 0%), Supporting Information: Figure 2.

## 4 DISCUSSION

We found a statistically significant association between the anterior–lateral electrode and increased cardioversion rate of AF compared with the anterior–posterior electrode in the overall analysis. Subgroup analysis showed no statistically significant difference between the anterior–lateral electrode and anterior–posterior electrode in patients who received five shocks, patients whose ages are less than 60 years old, and patients whose LA diameters are equal to and less than 45 mm. Subgroup analysis showed a statistically significant association between the anterior–lateral electrode and increased cardioversion rate of AF compared with the anterior–posterior electrode in patients who received less than five shocks, patients whose ages are equal to and above 60 years old, and patients whose LA diameters are more than 45 mm.

Our data suggest that the anterior–lateral approach is associated with higher cardioversion rates than the anterior–posterior approach. An anterior–posterior position was initially thought of as the optimal approach for AF cardioversion. Kirchhof et al. conducted a randomized clinical trial in 2002 that showed the superiority of the anterior–posterior approach in cardioverting AF. These findings were partially attributed to better electroanatomical convenience with the anterior–posterior electrode positioning, which would have greater proximity to the posteriorly located left atrium, creating a more efficient shock field gradient that is more efficient to terminate any fibrillatory activity in the left atrium.^7^ One downside of this trial is the use of monophasic shocks that were less efficacious than biphasic shocks in treating supraventricular arrhythmias.^10^

On the contrary, Schmidt et al. conducted a multicenter randomized trial of 468 patients, which concluded that the anterior–lateral approach was associated with higher cardioversion rates than the anterior–posterior approach.^11^ The underlying etiology remains unknown. They suggested that cardiac muscle is more sensitive to the direction of the shock vector, and hence more cardiac cells are cardioverted with this approach.^11^ However, more research is warranted to understand better the difference in outcomes between anterior–lateral and anterior–posterior approaches. Additionally, multiple small‐scale trials have shown no difference between the aforementioned techniques.^8^, ^12^, ^13^, ^14^

This significant discordance in outcomes can be in part attributed to multiple determinants associated with AF cardioversion. First, AF is associated with LA remodeling with subsequent LA enlargement. Nedios et al. conducted a prospective study of 115 patients with AF to investigate the impact of AF on LA remodeling. They used left atrial volume (LAV) and asymmetry index, ASI (which equals LA‐Ant./LAV), as markers for LA remodeling. They found that paroxysmal AF is more likely to have greater LAV and relatively higher ASI than the average population. Progression to persistent A.fib is more likely to have greater LAV with stable ASI. Eventually, long‐term persistent A.fib was found to have similar LAV to persistent A.fib with larger ASI.^15^

Moreover, the progression of A.fib is associated with poor outcomes after transcutaneous ablation.^15^, ^16^, ^17^ Surprisingly, our analysis reports better efficacy of anterior–lateral electrode positioning in patients with LA enlargement with a diameter greater than 45 mm when compared to anterior–posterior positioning. Additionally, the anterior–lateral position is associated with higher success rates in patients receiving less than five shocks. This denotes the superiority of the anterior–lateral position in managing this hard‐to‐treat subset of the A.fib population.

Second, a divergent patient population seems to be a valid prognostication factor in determining the safety and efficacy of various approaches. Our pooled analysis revealed that anterior–lateral position is more likely to attain higher success chances in patients aged 60 years old or more.

Third, it is essential to consider that some medical practices have been recently obsolete and are no longer clinical guidelines. One is the use of monophasic shocks in cardioversion which have been widely replaced with biphasic shocks that have shown optimal efficacy in managing different types of arrhythmias.^10^, ^18^ While Kirchhof et al. have used monophasic shocks and concluded more favored outcomes with the anterior–posterior approach, other randomized trials that have also utilized monophasic shocks showed no differing outcomes in either approach.^11^, ^19^ Standardized hand‐held steel paddles are associated with less intrathoracic impedance with a subsequent increase in intra‐thoracic shock field gradient compared to self‐adhesive electrodes. It is recommended to apply pressure on anterior–lateral electrodes for higher efficacy.^20^, ^21^

Given the current data, it is reasonable to consider the anterior–lateral position as a first‐line modality in AF cardioversion, given the relatively higher success rates, especially in elderly patients and those with enlarged left atrium indicating LA remodeling. Additionally, the anterior–lateral position has anatomical convenience and easier accessibility, particularly in critically ill patients.

Nevertheless, our study has some limitations. Variable protocols for cardioversion have been applied. While some papers followed the step‐up energy levels approach, others used initial high‐energy levels of 200 J and higher on failed attempts. We found some bias in deviation from the intended interventions, missing outcome data, and measurement of the outcome domains in some studies; however, we found no significant bias in the randomization process domain in the included studies. Lastly, our included articles had variable proportions of persistent and permanent AF, which are hard to treat and might alter the outcomes.

## 5 CONCLUSION

Our meta‐analysis revealed that anterior–lateral electrodeposition is more effective and better than anterior–posterior electrodeposition in external cardioversion of AF, particularly in patients who have received less than five shocks, are 60 years old or older, and have a LA diameter greater than 45 mm. More multicenter randomized clinical trials are warranted to support our findings.

## AUTHOR CONTRIBUTIONS

*Conceptualization*: Karam R. Motawea. *Research design, data collection, literature search, statistical analysis, and manuscript preparation*: Karam R. Motawea, Mostafa R. Mostafa, Mohamed Magdi, Mohamed Magdi, Hager Fathy, Sarya Swed, Mohamed M. Belal, Dina M. Awad, Rowan H. Elhalag, Nesreen E. Talat, Samah S. Rozan, Abdulqadir J. Nashwan, Naim Battikh, Bisher Sawaf, Mhd Kutaiba Albuni, Elias Battikh, Gihan M. Mohamed, Amr Farwati, and Hani Aiash: All authors read and approved the final manuscript. All authors take responsibility for all aspects of the reliability and freedom from bias of the data presented and their discussed interpretation

## CONFLICT OF INTEREST STATEMENT

The authors declare no conflict of interest.

## Supporting information

Supplementary information.

Supplementary information.

## Data Availability

All data generated during this study are included in this published article.
