# Supplementary figures and images for: Anteriolateral versus anterior–posterior electrodes in external cardioversion of atrial fibrillation: A systematic review and meta‐analysis of clinical trials
Source: Clin Cardiol. 2023 Feb 9;46(4):359–75. doi: 10.1002/clc.23987 (PMC10106664; doi:10.1002/clc.23987)

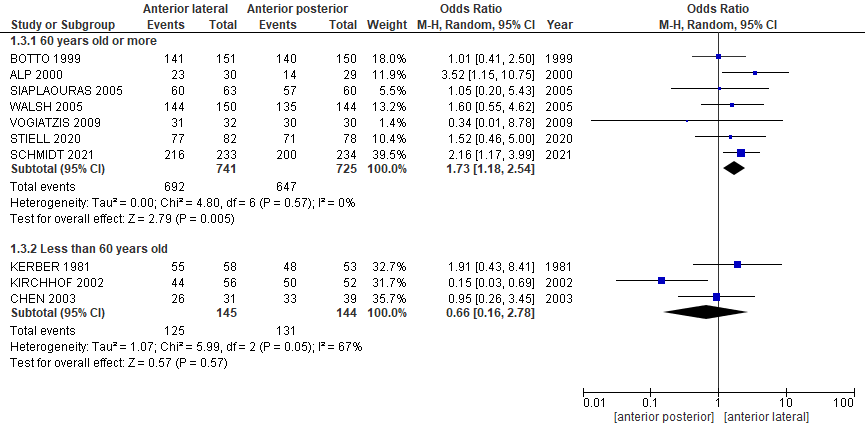
 Supplementary Figure 1. Age subgroup analysis

Supplement: Supplementary file 1 — Supplementary information. [file CLC-46-359-s001.docx]

Supplementary Figure 2. Left atrial diameter subgroup analysis


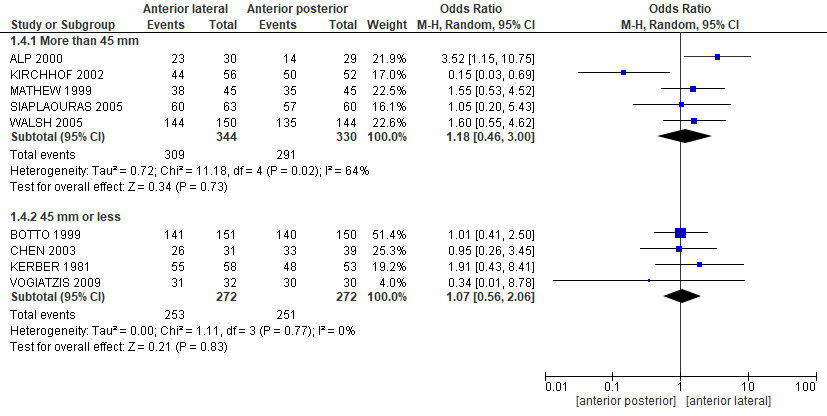

Supplement: Supplementary file 2 — Supplementary information. [file CLC-46-359-s002.docx]
